# The Impact of Living Situation on Healthcare Encounters for Individuals With Intellectual Disability

**DOI:** 10.7759/cureus.51156

**Published:** 2023-12-27

**Authors:** Calista Long, Eion Plenn, Samantha Acri, Cheryl Richardson

**Affiliations:** 1 Public Health, Penn State University College of Medicine, Milton S. Hershey Medical Center, Hershey, USA

**Keywords:** assisted living facilities, assisted living community, independent living, access to healthcare, intellectual disability (id)

## Abstract

Introduction

The living situation of individuals with intellectual disabilities (ID) has evolved throughout the years and ranges from living at home with family caregivers to group homes to independent living arrangements. Living situations can affect access to care and thus healthcare utilization seen by healthcare encounters for individuals with ID.

Methods

The researchers conducted a chart review of 112 patients to assess demographics, living situations, and healthcare encounters between 2019 and 2021. Living situation categories included independent, biological family, group home, home with other support, and others. Statistical analyses were conducted using R version 4.2.1 (The R Foundation for Statistical Computing, Vienna, Austria). Univariable analyses consisted of the Shapiro-Wilk test of normality, Kruskal-Wallis rank sum test, and pairwise Wilcoxon rank sum test with multiple comparisons correction using the Bonferroni method. Statistical testing for multivariable analysis included the Kruskal-Wallis rank sum test, Spearman’s rank correlation, and the negative binomial model.

Results

Results showed a statistically significant difference in median total encounter value between independently living individuals with ID compared to all other living situations, Χ^2^ = 4.230, df = 1, p-value = 0.040. Additionally, there is a significant association between medication count and total encounter count, rho = 0.341, S = 154322, p-value < 0.001.

Conclusion

The study showed that individuals with ID who live independently have fewer healthcare encounters compared to all other living situations. This may be due to various factors such as increased autonomy and free choice, increased barriers to healthcare, or better overall health requiring less medical attention in independently living individuals with ID.

## Introduction

The term intellectual disability (ID) is used to describe individuals with significant limitations in intellectual functioning and adaptive behaviors that originate before age 22, thus influencing social and practical skills [1]. It is an ever-evolving discussion of how to best care for those with ID. One factor that plays a large role in this population’s care is living arrangements.

In prior decades, individuals with ID lived at home and received most of their care from family members and members of the community [2]. After that, living arrangements were primarily in educational institutions that then became medically based [2]. In recent years we have seen a return of integration of this population back into society and more community-based arrangements. This new model of small-scale community-based housing arrangements leads to better outcomes in categories such as community presence and participation, social networks and friendships, family contact, self-determination and choice, and quality of life [3]. Despite these improved outcomes, transitioning from long-stay psychiatric hospitals to community-based living arrangements also has negative impacts such as placing a greater burden of care on primary care providers in the community [4].

Community-based living has resulted in new challenges for healthcare access and utilization in a population that already has poorer health outcomes compared to the general population. Individuals with ID suffer from avoidable medical harm events, delays, and omissions of care such as inadequate provision of basic nursing care, misdiagnosis, delayed investigations and treatment, and non-treatment decisions [5]. Adults with ID experience higher rates of hospitalizations and are at higher risk for preventable emergency admissions for ambulatory-sensitive conditions such as lower respiratory and urinary tract infections [6]. They also experience higher rates of chronic health problems and poorer overall health compared to those without ID. These health problems are specific for people with ID, both in general and living in the community in particular [7]. Not only does this population have increased mental illness and physical illness rates, but studies over the last four decades have consistently shown that individuals with ID die prematurely. It is estimated that life expectancy is reduced by up to 20 years in people with ID and was found to be 50.4-58.7 years of age on average [8,9].

There are many proposed factors by which these health discrepancies occur. Health risk behaviors such as poor diet, lack of physical activity, and underuse of health care are some proposed factors that may result in individuals with ID experiencing a higher incidence of being overweight and sedentary, and higher rates of arthritis, asthma, diabetes, high blood pressure, high cholesterol, cardiovascular disease, and stroke [10]. Additional factors resulting in poorer health outcomes and health disparities for individuals with an ID include decreased presentation to their general practitioners leading to a lack of identification of treatable conditions, little to no guidance regarding their health through measures such as nutrition and exercise, and diagnostic overshadowing that can lead to inadequate workup and treatment for conditions [8]. People with ID also experience lower rates of screening and more difficulty accessing services, compared to people without disabilities [11]. As the living situation of adults with ID has transitioned to more community-based housing, the question remains how this shift has affected their access to and utilization of health services and thus health disparities.

Regarding certain illnesses that adults with ID experience, sources have found important differences between different living arrangements depending on the level of formal support available and the stage of deinstitutionalization. There are identifiable deficits in variables related to medical health promotion measures such as vaccinations, cancer screenings, and medical checks in family home settings and independent living arrangements. This shows the importance of primary health programs that guarantee access to quality healthcare which includes preventative health actions of vaccination programs, systematic health checks, specific screenings, and nutritional controls in adults with ID in more community-based living arrangements [12].

While there are many factors that influence healthcare and health disparities for individuals with ID, living arrangement is one that has been seen to continuously evolve over time. Thus, the purpose of this study is to explore how different living situations impact the number of medical encounters for individuals with ID.

## Materials and methods

Retrospective chart review sample selection process

**Figure 1:**
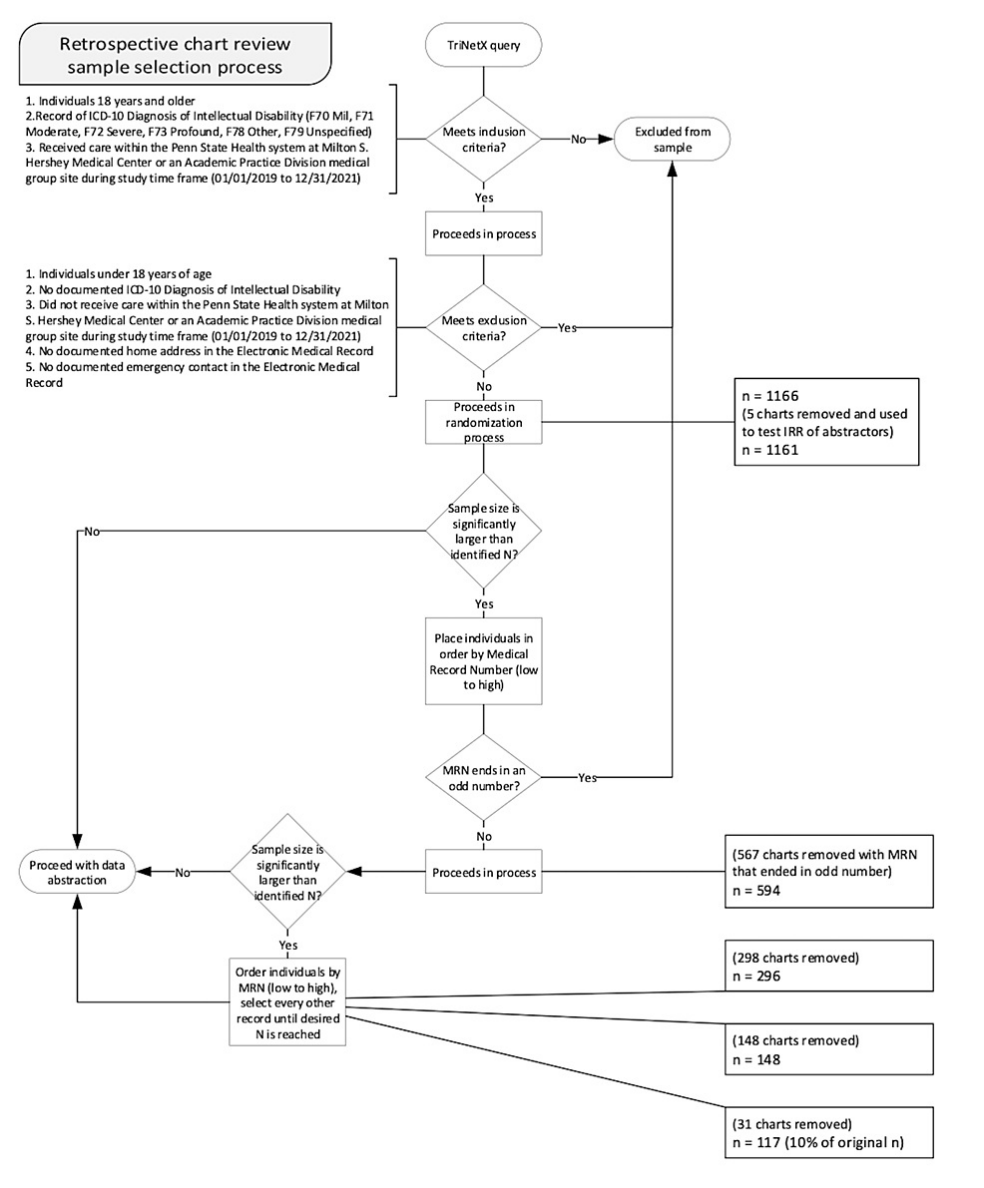
Retrospective chart review selection process (flowchart)

A retrospective chart review was used to determine if the living situation of individuals with ID affects the number of healthcare encounters. Using patients within a regional health system (identified via TriNetX (Cambridge, MA, USA)), data were collected for the years 2019, 2020, and 2021 to study access patterns among individuals with ID. Study data were collected and managed using REDCap (Vanderbilt University, Nashville, TN, USA) electronic data capture tools hosted at the study site [13]. The study followed all required ethical and consent protocols per the Institutional Review Board at Pennsylvania State University and was deemed exempt. Inclusion criteria were as follows: (1) Patient is 18 years and older; (2) Patient has record of ICD-10 Diagnosis of Intellectual Disability (F70 Mild, F71 Moderate, F72 Severe, F73 Profound, F78 Other, F79 Unspecified); and (3) Patient received care within the health system at either the academic medical center or the academic practice medical group sites during study time frame (01/01/2019 to 12/31/2021). Exclusion criteria included: (1) Individuals under 18 years of age; (2) No documented ICD-10 Diagnosis of Intellectual Disability (F70 Mild, F71 Moderate, F72 Severe, F73 Profound, F78 Other, F79 Unspecified); (3) Did not receive care within the health system at either the academic medical center or the academic practice medical group sites during the study time frame (01/01/2019 to 12/31/2021); (4) No documented home address in the Electronic Medical Record (EMR); and (5) No documented emergency contact in the EMR. The sample size was reduced to a size that met the needed power for the study yet allowed for full abstraction. The sample size was narrowed to be representative of 10% of the total population (Figure 1). Once the appropriate sample size was obtained, the study proceeded with data abstraction and coding for living situations.

Procedure

**Figure 2:**
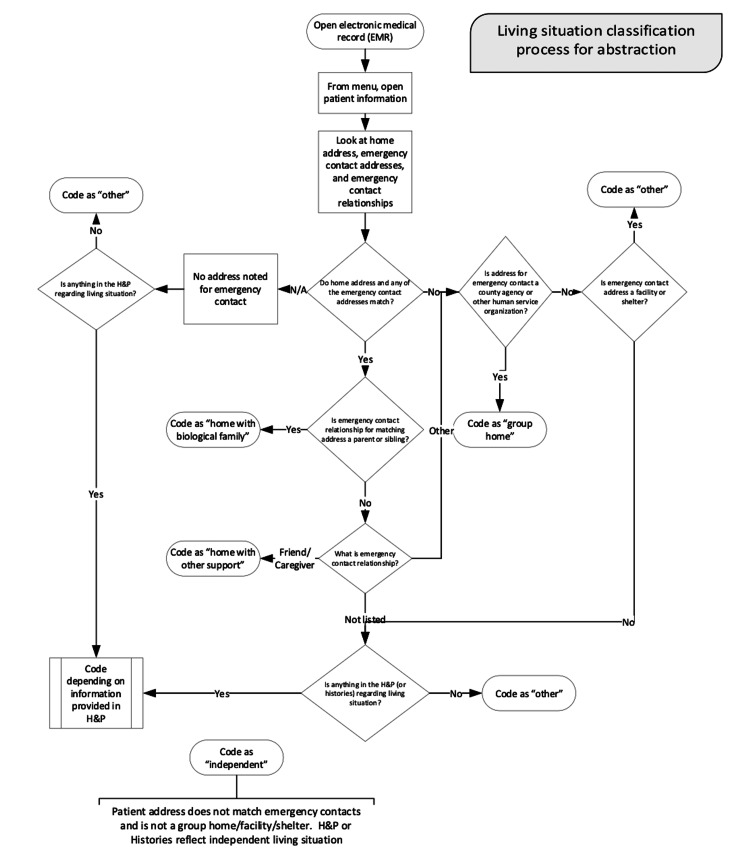
Living situation classification process for abstraction (flowchart)

Patient medical records were reviewed retrospectively for both demographic factors and medical information by two independent researchers (CL and EP). Demographic information included date of birth, gender, race and ethnicity, level of ID, and living situation. The living situation was coded into one of six categories based on the documentation of the patient’s home address, emergency contact address, and emergency contact relationship (Figure 2). These categories included independent, home with biological family (individual resides with parents or siblings), home with other support (individual resides with a consistent caregiver who is not the parent or sibling), group home (support provided by staff who may change), and other (used only if there is an address on file and the living situation does not fit the definition of the other categories). Patients without a documented home address were excluded. Figure 2 details the living situation classification process. Information abstracted from patients’ EMR included primary care provider; the number of primary care provider visits in the last three years (January 2019 to December 2021); the number of emergency department (ED) visits in the last three years (January 2019 to December 2021); the number of acute hospitalizations in the last three years (January 2019 to December 2021); and the total number of medications. If the patient was prescribed an antipsychotic, the name and class (typical or atypical) were documented as well as the presence or absence of a corresponding mental health diagnosis and if the patient received mental health services in the last year.

Statistical analysis

Following chart abstraction, all analyses were conducted using R version 4.2.1 (The R Foundation for Statistical Computing, Vienna, Austria) to determine the relationship between the independent variable (living situation) and the dependent variable (acute hospitalizations) when accounting for covariables. Independent analysis of the results was done by a biostatistician to help reduce bias. Prior to analysis, one record was excluded due to missing age, and four records were excluded due to having zero encounters during the three-year lookback. All descriptive statistics were reported as percentages or medians since the data were not normally distributed. Statistical tests used for univariable analysis included the Shapiro-Wilk test of normality, the Kruskal-Wallis rank sum test, and the pairwise Wilcoxon Rank Sum test with multiple comparisons correction using the Bonferroni method. Statistical testing for multivariable analysis included the Kruskal-Wallis rank sum test, Spearman’s rank correlation, and the negative binomial model.

## Results

The chart review yielded 112 unique patients for analysis after the exclusion of one record due to missing age and four records due to having zero encounters during the three-year lookback.

Patients had a mean (SD) age of 39.9 (17.8) years and were 53% male and 72% white. The ICD-10 code for the majority of patients was F79 unspecified ID. The primary living situation was with biological family at 55.4% followed by independent at 17.0%, group home and other both had 9.8%, home with other support at 5.4%, custodial care at 1.8%, and shelter at 0.9%. Additional descriptive statistics are presented in Table 1.

**Table 1:** Demographic data for individuals with intellectual disability Demographics including gender, ethnicity, race, disability level, living situation, and anti-psychotic medications for patients with intellectual disabilities within the health system (identified via TriNetX), data was collected for the years 2019, 2020, and 2021.

Feature	Category	Value
		Median (IQR)
	Patient Age	35 (33) years
	Patient Age Standard Deviation	17.8
	Visits to PCP Office	0 (4)
	Visits to Specialist	6 (14)
	Emergency Encounters	0 (1)
	Hospital Admissions	0 (1)
	Unique Medications	8 (10)
		Count (Percentage)
Gender	Female	53 (47.3%)
	Male	59 (52.7%)
Ethnicity	Hispanic/Latino/Spanish origin	7 (6.2%)
	Not Hispanic/Latino/Spanish origin	100 (89.3%)
	Unknown	5 (4.5%)
Race	American Indian or Alaska Native	1 (0.9%)
	Asian	5 (4.5%)
	Black or African American	12 (10.7%)
	White	81 (72.3%)
	Other/Unknown	13 (11.6%)
Disability Level	F70 Mild	15 (13.4%)
	F71 Moderate	12 (10.7%)
	F72 Severe	4 (3.6%)
	F78 Other	5 (4.5%)
	F79 Unspecified	76 (67.9%)
Living Situation	Biological family	62 (55.4%)
	Custodial care	2 (1.8%)
	Group home	11 (9.8%)
	Home with other support	6 (5.4%)
	Independent	19 (17.0%)
	Shelter	1 (0.9%)
	Other	11 (9.8%)
Anti-Psychotic Med(s) Prescribed	No	69 (61.6%)
	Yes	42 (37.5%)
	Unknown	1 (0.9%)

Univariable analysis

**Figure 3:**
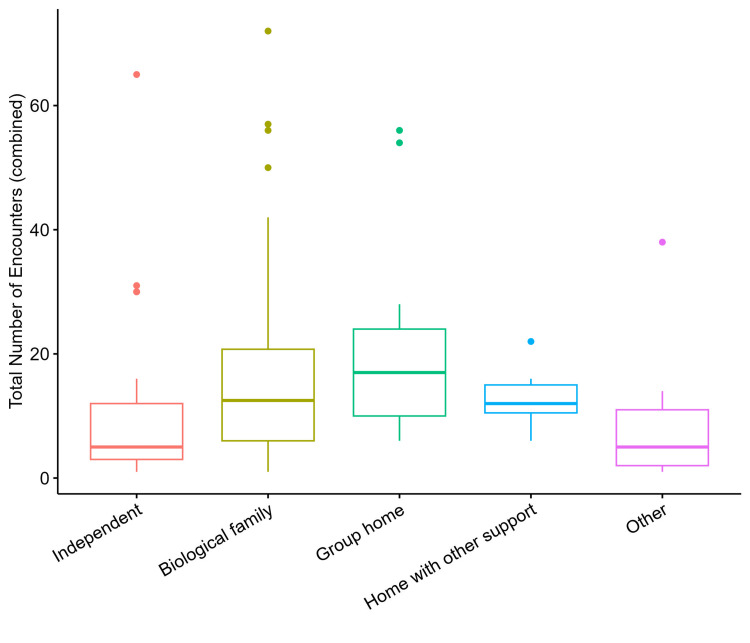
Healthcare encounter count by living situation Total encounters are not normally distributed as confirmed by the Shapiro-Wilk test of normality (overall and by living situation group). Per the Kruskal-Wallis rank sum test, Χ^2^ = 12.846, df = 4, p-value = 0.012, the median total encounters value statistically differs among living situation groups suggesting at least one is different than another.

**Figure 4:**
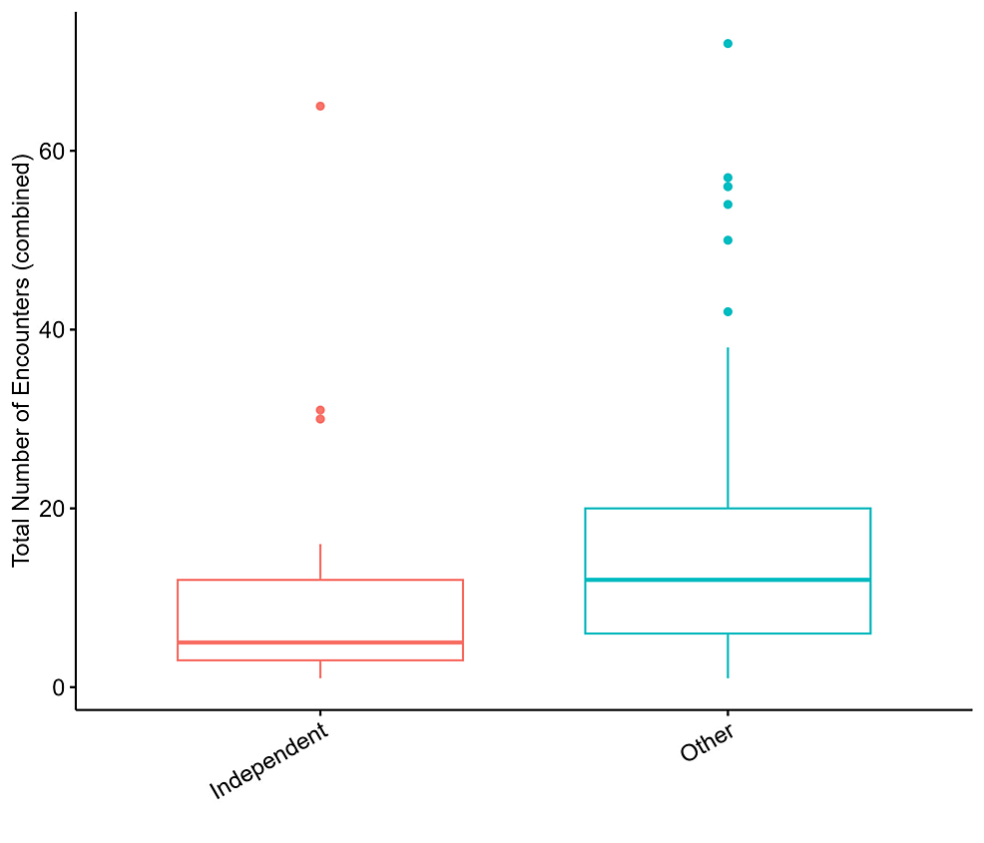
Healthcare encounter count by the living situation with a condensed grouping of Independent vs. all others The difference in median total encounter value is significant, Χ^2^ = 4.230, df = 1, p-value = 0.040, per the Kruskal-Wallis rank sum test.

When looking at healthcare encounter count by living situation, group home had the highest median encounter count at 17, followed by biological family and home with other support at 12, and independent and other at 5 (Table 2). Preliminary testing for statistical significance between living situations groups and total healthcare encounters was positive with Χ^2^ = 12.846, df = 4, p-value = 0.012 (Figure 3). When evaluating with pairwise comparisons to determine which groups differ from one another according to pairwise Wilcoxon Rank Sum tests after correcting for multiple comparisons using the Bonferroni method, there was no difference between any of the groups. The closest association was for group home vs. other which was approaching significance with a p-value of 0.051. When condensing the groupings and comparing only two living situation groups of independent vs. all others, there was a statistically significant difference in median total encounter value Χ^2^ = 4.230, df = 1, p-value = 0.040 per the Kruskal-Wallis rank sum test. The median healthcare encounter was 5 for independent living and 12 for all other living situations for patients with ID (Figure 4).

**Table 2:** Healthcare encounter count by living situation Total encounters are not normally distributed as confirmed by the Shapiro-Wilk test of normality (overall and by living situation group). Per the Kruskal-Wallis rank sum test, Χ^2^ = 12.846, df = 4, p-value = 0.012, the median total encounters value statistically differs among living situation groups suggesting at least one is different than another.

Living Situation	Number of Patients	Minimum	Quartile 1	Median	Quartile 3	Maximum
Independent	19	1	3	5	12	65
Biological family	62	1	6	12	20	72
Group home	11	6	10	17	24	56
Home with other support	6	6	10	12	15	22
Other	14	1	2	5	11	38

Multivariable analysis

To explore the relationship between living situation and healthcare utilization while controlling for other factors, a multivariable negative binomial model was used. A single feature (medication count) besides the living situation was individually associated with total encounter count (Spearman’s rank correlation test statistic = 0.341, p-value < 0.001, 95% confidence interval [0.161, 0.499]), so the interconnectedness of the living situation, medication count, and healthcare utilization was of particular interest. The multivariable analysis did not reveal any new understanding. Both living situation and medication were predictive of total encounter count, but an interaction term between the two was not significant. These results suggest that living situation individually predicts healthcare utilization and that the relationship is not confounded by any other measured variables.

## Discussion

The study sample showed a vast majority of subjects living in community-based housing arrangements, primarily with biological families (Table 1). This is consistent with the shift toward community-based living that has been seen since The Americans with Disabilities Act of 1992. Antipsychotic medications were prescribed for 61.6% of subjects and there was a significant association identified between medication count and total encounter count (rho = 0.341, S = 154322, p-value < .001) (Table 1). This may indicate that patients who receive more frequent medical care through healthcare encounters do so for access to medications. Likewise, it could highlight a disparity in patients with ID and fewer healthcare interactions not receiving medication that they might benefit from. Overall, the significance between medications and the frequency of healthcare encounters could suggest an issue with access to care that individuals with ID face.

The identified statistically significant difference in healthcare encounter count and living situation was for independently living individuals with ID compared to all other living situations. The identified relationship revealed that independently living individuals with ID have less median healthcare encounters with an average of 5, compared to all other living situations with an average of 12 (Figure 4). This might suggest that individuals with ID who live independently have less access to healthcare than those residing with others, thus resulting in fewer healthcare encounters. The cause of this may be due to many factors. First, individuals with ID may have certain barriers that prevent them from receiving healthcare which may include transportation, communication barriers, finances, and poor understanding of health needs. Additionally, various studies have demonstrated that independent living results in increased autonomy and free choice [14,15]. Perhaps, independently living individuals have more autonomy and choose not to attend medical encounters whereas those who have less autonomy, are instead receiving healthcare encounters by family, caretaker, or facility influence or requirements. For example, studies have shown that individuals with ID who live with family or other caretakers, demonstrate additional health service requirements such as going to the ED due to mental health problems and ensuing psychiatric emergencies [16].

An alternative view is that rather than choosing not to seek medical care, independently living individuals with ID instead have more difficulty accessing medical care as research has shown that people with disabilities experience more difficulty accessing services compared to people without disabilities [11]. Barriers to healthcare, such as transportation challenges, may result in independently living patients with ID not being able to attend medical appointments. An identified factor resulting in poorer health outcomes is decreased presentation to general practitioners leading to a lack of identification of treatable conditions [8]. The effects of decreased access may be even more profound in this population as individuals with ID already experience more physical and mental health conditions than people without ID and may need even more health checks than individuals without ID [8]. This could result in even further health disparities and negative health outcomes for independently living individuals with ID, as evidence has shown that healthcare needs might not be adequately met in the community [17].

Another possible factor is that poor health provider preparedness in treating this population and poor healthcare experiences leads to low follow-up and decreased willingness to seek medical care from individuals with ID who live independently. A review of eligible papers revealed that despite 20 years of research and government initiatives, individuals with ID continue to have poor hospital experiences [18]. Issues such as inadequate provision of basic nursing care, misdiagnosis, and delayed investigations and treatment are just some of the safety issues that individuals with ID experience with receiving healthcare [5]. Additionally, many people with ID have described hospital staff as having negative attitudes and a lack of skills and knowledge regarding their needs [19]. The disparities in healthcare could be due to healthcare providers not being adequately equipped to treat this population.

An alternative view is that perhaps independently living individuals with ID are healthier and thus do not require healthcare encounters as frequently as other living situation groups. A study based in Sweden by Mrayyan et al. showed that individuals with ID who live in special housing such as a group home experience more inpatient and unplanned visits [19]. An explanation for this may be that individuals with ID who live in specialized housing such as group homes have poorer health than those who live independently [19]. Our data revealed a significant relationship between the number of medications and hospital encounters, if individuals with ID who are in special housing are more severely ill, they may be prescribed more medications and experience more healthcare encounters.

Limitations of this study include a small sample size, particularly regarding medical encounters as well as restriction to only one health system which may reduce generalizability. Sources of bias include nonblinded chart review for data abstraction and lack of a second reviewer to re-abstract to improve accuracy. Furthermore, our study assumed living situation and healthcare encounters were two distinct variables, when in fact there are other factors that may play a role in this relationship such as relative state of health. Additionally, utilizing demographic information within the EMR to determine living situations may result in inaccuracies, and thus examination of medical documents or utilizing survey methodology to determine living arrangements may be more accurate.

The strengths of this study include the investigation of a topic and relationship with minimal existing literature. The findings provide important insights into unique challenges that individuals with ID may face based on their living situation, and how this may create barriers to accessing medical care. Implications include advocating for improved screenings, primary care access, and community support for individuals with ID who live independently. Additionally, it speaks to the importance of clinicians being knowledgeable of the intricacies of providing adequate healthcare to this population, and how poor healthcare experiences may play a role in the lack of follow-up or subsequent healthcare encounters. Future directions should focus on exploring and addressing barriers that result in independently living individuals with ID receiving less healthcare encounters. Additional future directions include replicating this study with a larger sample size and geographic diversity to build upon the current findings. Additionally, further exploration of the impact of demographic factors such as race, gender, and socioeconomic status on healthcare encounters and living situations for individuals with ID would provide further investigation of this relationship.

## Conclusions

This study explored the relationship between the living situation of individuals with ID and the frequency of healthcare encounters. The analysis found a statistically significant difference in median total encounter value between independently living individuals with ID compared to all other living situations, Χ2 = 4.230, df = 1, p-value = 0.040, showing that independently living individuals with ID have less healthcare encounters than other living situations. This may be due to individuals with ID who live independently having increased autonomy and free choice, increased barriers to healthcare, or better overall health requiring less medical attention in independently living individuals with ID. Additionally, this study demonstrated that a vast majority of individuals with ID live in community-based housing arrangements, with biological family being the highest percentage of the living situation at 55.4%. There was also an identified significant association between medication count and total encounter count (rho = 0.341, S = 154322, p-value < 0.001) which may point to patients who are receiving more regular medical care and healthcare encounters having greater access to medications.
